# Introductory to the 46th Annual Course of Instruction in the Hahnemann Medical College of Philadelphia

**Published:** 1893-12

**Authors:** B. Frank Betts

**Affiliations:** Professor of Gynecology


### INTRODUCTORY TO THE 46TH ANNUAL COURSE OF INSTRUCTION IN THE HAHNEMANN MEDICAL COLLEGE OF PHILADELPHIA.

B. FRANK BETTS, M. D., PROFESSOR OF GYNECOLOGY.

The opening of this, the forty-sixth annual course of lectures, is an event of particular interest to the physicians and students of our profession ; for as the oldest homœopathic college in the world completes another cycle in its history, we naturally look about us for evidences of such an improvement in methods of instruction as will secure to those who are to form an integral part of the profession in the near future the opportunity to acquire such proficiency in their medical studies as will enable them to keep pace with the rapid advancement made in medical science.

The practical clinical instruction of students has always constituted an important part of the curriculum of this college, and I am glad to be able to report that the out-patient department of the hospital has been much enlarged by the addition of two wings to the building, so that better accommodations can be afforded both patients and students, giving us at least one-third ($\frac{1}{3}$) more space than before.

This is needed, for the large class of students in attendance last year, with the number of patients seeking medical and surgical treatment crowded the building, although the surgical department had been moved into the basement of the hospital building, along with the gynecological department before the opening of the last session.

You are probably aware that we divide the class into sections and that each section consists only of such a number as may come into direct communication with clinical cases treated by the clinical instructors in the several departments of the dispensary.

These small classes are assigned to the different departments in rotation, and we aim to afford them an opportunity to witness methods of diagnosis and treatment applicable to forms of disease most likely to be met with in the ordinary course of a physician's practice.

During the past year thirteen thousand three hundred patients have been treated in the out-patient department of the hospital and three thousand five hundred and seventy in the hospital proper.

In the public wards and in the out-patient department, most of the charitable work of the institution is done. This part of the hospital is an important adjunct to the college in the education of students, but in the private rooms, patients are treated by the physicians and surgeons of their own selection without having any communication with the public wards or clinical departments. These are in nowise available for the clinical instruction of students.

The property adjacent to the hospital on Fifteenth Street, purchased for the purpose of establishing an obstetrical department in conjunction with the other departments of the hospital is still unimproved; but plans are being prepared and the erection of an obstetrical pavilion will be commenced as soon as sufficient funds are secured.

In order to give students more time to pursue their college studies, the annual session has been lengthened to seven months, so that instead of closing the session the last of March, it will continue until the last of April. The spring course of lectures will be abolished, as the regular session will extend into the time usually devoted to it. The faculty have under contemplation the establishment of a short post-graduate course, for practitioners, which will follow directly upon the close of the regular session. The week of preliminary lectures before the opening of the course has been dropped.

After this year, the four years' graded course will be made obligatory, so that the students matriculated next fall will be obliged to devote *four years* to the study of medicine before they are graduated.

By recent legislative enactments in several of the States, Medical Licensing Boards of Medical Examiners have been established. In Pennsylvania three separate Boards of Examiners are provided for, the Allopathic, the Homœopathic, and the Eclectic Boards.

They are all under the control of a Medical Council composed of the Lieutenant-Governor with other State officers together with the President of the State Board of Health, and the President of the three Boards of Medical Examiners.

This Council supervises the examinations conducted by the three Boards and issues licenses to practice to those who have successfully passed the examination by the Boards. The Medical Council also issues licenses to such applicants as present satisfactory and properly certified copies of licenses from other State Boards or bodies authorized to grant them.

The Medical Examiners of Pennsylvania will be appointed by the Governor in January next and annually thereafter. Students who are graduated after the first of July, 1894, must be examined and licensed before they can be registered and permitted to practice in this State. The questions submitted to the applicants before the three Boards are to be the same with the exception of questions in Therapeutics, Practice in Medicine, and Materia Medica, which latter, must be in harmony with the teachings of the School of Practice selected by the candidate.

The same standard of qualifications is exacted of the candidates from each school of practice.

By this enactment the responsibility of the colleges is materially increased. Their standard of qualification must conform to the standard of the licensing power or else the diploma will be of no value. Furthermore, the preliminary examination of students must be such as will leave little doubt in the minds of the faculty as to the ability of the candidate to acquire the knowledge necessary to pass the final examination satisfactorily, otherwise the student's time and means may be wasted.

According to the present arrangement the issuing of a diploma to a student is of little account without it carries with it evidence of such acquirements in its possessor as will lead him to entertain a reasonable expectation of passing the examination of the Medical Board. If the diploma has been granted by a high-grade college it should meet all of these requirements.

I am glad to be able to state that in New Jersey, where the examination is conducted by a mixed board, and all knowledge of the applicant's school of practice is ignored, no graduate from the Hahnemann Medical College, of Philadelphia, has been denied a license since the law went into effect two years ago, although out of a total of one hundred and forty-three examinations, thirty-two have been rejected from other colleges.

Furthermore, from the annual reports I find among eighteen (18) examinations of graduates from the University of Pennsylvania, the oldest and one of the best endowed medical schools in this country, the general average attained was 83.50. From the twelve (12) examinations of graduates from our college the general average was 81.90, but 1.60 below the former. From eight (8) examinations of graduates of the Homœopathic Medical College of New York the general average was 75.96, and from the twenty-one (21) examinations of graduates from the Jefferson Medical College, of Philadelphia, the general average was 74.99 ; an average of 75 is necessary to pass successfully. My object in mentioning these facts is to prove the statement that the standard of qualification maintained by the Medical Licensing Boards is not likely to be above that of the best medical schools, and that the general average of graduates from this school compares very favorably with that of the best schools in the country.

But, gentlemen, I am far ahead of the theme that interests me most to-night. It is not by the possession of a diploma or a license to practice that you are to secure success in your professional life. Your presence here assures me that you have some knowledge of the efficacy of homœopathic treatment, and that it is your aim to acquire an understanding of this system of practice at this institution.

By careful study and observation, you expect to become skilled in the art of healing. This constitutes a physician.

Other themes may interest you and claim attention, but the object of paramount importance *must be,* proficiency in the art of healing.

During your college days your attention will be directed, first, to the study of the human organism in health; then, to the human organism—sick; for it is necessary that you should know the well man, well, before you can understand the sick man well. Upon your knowledge of Anatomy, Physiology, Chemistry, and Histology therefore much will depend.

Later on your attention will be directed to the morbid changes produced by disease. The signs and characteristic features of the different diseases; their diagnosis and treatment must be studied—and just here the paths trodden by the allopathic and the homœopathic fraternity diverge, and it should interest you to know in what important respects they differ.

The opposite school bases its medical treatment upon a knowledge of those morbid disturbances and changes wrought in the organs and tissues of the body by disease as is obtained by means of physical explorations and symptomatic indications. Whilst the distinctive features of Hahnemann's plan as explained in *The Organon,* consists in basing medical treatment upon a knowledge of morbid disturbances in the *vital or life force* throughout the whole organism, made manifest to us by symptoms and physical signs.

It was to a fuller recognition of the *vital power or life principle* in the individual that Hahnemann directed attention. He never claimed to have discovered this dynamic force, for it had been recognized by medical men ever since Althenaens taught the active principle of "pneuma" as the basis of existence in the earliest days of the Christian era. Neither could he describe it, except as an imponderable, invisible, all-pervading power animating, shaping, and controlling tissue changes within the organism, until the end, when its influence stops, and in death purely chemical laws gain control.

Into man, fashioned from the elements of the earth, typified in clay, the Creator breathed the breath of life and this life principle has been transmitted from individual to individual ever since. The ovum contains it, whilst it is an integral part of the mother's living organism and the sperm vesicle is endowed with it. Without this principle in both of these organisms there can be no growth—no new formation. When this vital principle present in every part of the living organism is confronted by certain influences which we term morbific or such as are conflicting, encumbering and combatting, the tendency is sickward, or away from a perfect state of health, and symptoms of disease are complained of.

From hereditary weakness in a part or in all parts of the organism the individual becomes more susceptible to the influences of morbific agents. Unhygienic surroundings, over-work, excesses of all kinds weaken the vital force and the disease-engendering influence gains the ascendency.

It is undoubtedly due to the weakening of this vital energy that disease becomes more virulent in some cases than in others, and not as some suppose to an excess in the quantity of the morbific agent.

A man may reside in a pest-house for months and not contract disease, but let him get demoralized, frightened, and run down in health and it will be very likely to gain an ascendency over the health-dealing influences.

The ptomaine that is developed in the tissues from the presence of microbia is inert if the system is in a perfect state of health, but when tissue metamorphosis is weakened it becomes a most virulent element of morbific power, the addition of a single chemical atom to one of its component parts as previously existing is all that is necessary to change it from an inert to a toxic agent in many cases.

Degradation of tissue results in the development of noxious elements out of inert substances under such circumstances.

Kijaintzen of Kiew has demonstrated the presence of a special and peculiar poisonous ptomaine developed in the tissues in consequence of extensive scalds or burns upon the surface of the body.

This substance when isolated from the organism and injected into another person is capable of producing depression of temperature, weak heart action, slow, shallow breathing, diarrhœa, vomiting, somnolence, apathy, stupor, etc.—symptoms characteristic of persons dying from extensive scalds and burns—so that it seems probable that when death is not due to sudden shock it may result from the development of a poisonous principle in the tissues formed from lowered vital action before extraneous septic infection occurs. It is well known that emotional influences act upon the glandular secretions to vitiate them, as when a nursing mother sickens her child by feeding it from the breast after she has been violently angered. It is also said that anger changes the character of the saliva in some mysterious way so that it produces serious results when introduced underneath the skin by biting.

As an illustration of the wonderful influence of this life principle *in health* I might allude to the production of the normal temperature in the human organism. Whether this function is under the immediate control of a separate nerve centre or not, the production of heat up to a certain standard as well as its dissipation at other times under varying temperatures of the surrounding media is a most wonderful illustration of this controlling influence in the organism which amounts almost to intelligence. Imperfect control over tissue metamorphosis not only favors the development of toxic agents or poisonous principles but leads to the development of fever in acute cases, and to new growths in the tissues such as tumors and mal-formations under other conditions. In the latter case we see only the effects produced by the change in the vital force, yet long before the tumor can be detected or the inflammatory swelling develops the system is disturbed and symptoms are complained of. These may seem to have had no connection with the pathological product which we are able to detect later, for often these disturbances are trivial and apparently require no particular treatment, yet from the fact that children subjected to homœopathic or symptomatic treatment during their growth and development are less prone to develop these tumors in after life than others, we may infer that much that might have developed has been prevented.

A person may suffer from intense neuralgia without any pathological change in the nerve trunk or its centre of origin, and yet in time the tissues supplied by it, waste, the hair turns gray, the skin may become inflamed or atrophied. If we can cure the symptomatic evidence of disturbance in the nerve we prevent the after effects of its functional derangement.

The change from static to a dynamic condition of the nervous system by imponderable, undiscovered, and in every way inappreciable influences is well illustrated in some cases of spasmodic asthma in which an attack may be induced by the slightest change in telluric influences as by a journey from one point to another only a few miles distant.

The influence of malarial poisoning upon the vital forces of the organism is most remarkable, yet this poison is imponderable, invisible, and absolutely unknown except from its effects upon the vital force. I think that these illustrations are sufficient to prove that medical treatment should not be based upon a knowledge of pathological changes in the organs and tissues of the body or the evidences secured by physical exploration alone, but upon symptomatic indications afforded by disturbances in the vital force. It is the change effected in the vital energy which gives us a complete picture of the diseased condition to which we must apply our art of healing. As this change extends to all parts of the organism—even the mental symptoms of a sick man are often of importance. To this changed condition, this deviation from health, we apply remedies according to the homœopathic law.

When disturbances in the vital activity of the organism have resulted in the development of *new* growths with structural elements *unlike* those of the tissues in which they are found, constituting malignant tumors, all medicines fail to effect curative results. These conditions are beyond the control of normal, healthy vital action. Methods of diagnosis have to be studied and carefully pursued to enable us to distinguish the character of these diseases, and surgical means have to be resorted to, to effect the prompt removal of all such obstacles as interfere with the application of *our* art of healing; but through all our experience in the treatment of disease we must be ever mindful of the presence of that vital force which is in every living cell and tissue element, and constitutes the only real difference between a human being alive and the same being dead. Its action may be perverted, but until it ceases in death we may aim to influence it for the improvement of tissue metamorphosis by homœopathic medication.

An eminent authority we all respect, one who is known the world over, remarked in an address before a number of medical students of the opposite school as follows :

" The materialistic conceptions students imbibe often make them look upon man as a mere machine on which to work with knife and saw, and into which he is to cast with more or less thought his drugs, but the soul of man dwells in this house of clay," and the principles of life build and fashion it at every stage of its existence ; " we deal not with the *bodies* of men alone ; woe to them and their physicians if this be their definition of medicine. Men's souls, men's lives, their secrets and aims, come all within the range of our perception."

The same author says : " Alas ! how little are the counted gains of the long generations of our guild who have faced fairly the problem of life." I might quote many paragraphs from the works of standard authors of the other school to show the dissatisfaction felt with the old methods of treating disease. " We dislike to confess," says one, " that there is *no* direct treatment for internal pathological conditions, and we therefore eagerly seize upon whatever is presented to us for trial, no matter whence it comes. The antipyretics were a great inovation at one time, now the same who used them then, say, ' don't touch them.' We used to believe that the salicylates were almost specific for rheumatic poison, now we learn that they act as anæsthetics for rheumatic pain, but have no permanent curative effect, indeed some claim that they increase the tendency to renewed attacks. Binoxide of manganese is lauded for amenorrhœa ; soon many try it, but are disappointed and it is dropped."

" In the treatment of cholera everything has been suggested, but little scientific advance has been made." Russia has lost over two hundred thousand of her inhabitants from this disease during the last epidemic, and in Austria the people have resisted the treatment which did not cure a single member of whole families, whilst patients hidden away recovered, so that it became necessary to afford military protection to physicians practicing in the infected districts.

Bacteriology has afforded us the most wonderful facilities for the *prevention* of disease, and nothing has been discovered that has shed more honor upon the medical profession than this. But we quote from the *Medical Record*, of New York, which says, " whilst we are as much interested in the discovery of new germs as in the discovery of a new comet, or latest unearthed mastodon, which are interesting as facts, we find they have no special value to us in curing disease, for no parasiticide has been discovered that is absolute and certain in its results when administered to man."

Tuberculin has proved inefficient, and many such experiences have been delusive, so that the profession has had to turn back to old methods of treatment again. The constant association of microbia with any or all such diseases is but one fact in connection with them; and such a discovery is to be regarded merely as a step forward, to be followed by others, which will represent not only an advance in our knowledge of the nature of disease, but of the processes of the hidden vital power as well. Disappointment in methods of treatment must always follow, without we take into consideration the fact that conditions of ill-health are dependent upon disturbances in the vital powers of the organism, and disturbed vital action must exist before microbia can produce their most serious toxic influence. In animals subjected to experimentation it is developed when they are wounded or subjected to unnatural influences. If their powers of resistance are normal they can combat the influence of microbia. With diminished powers of resistance the same phenomena may sometimes develop without the presence of microbia.

*Pus* is not always to be regarded as of *infectious* origin, for there are pyogenic agencies, like Petroleum, Turpentine, and Croton Oil, which introduced into the body under certain conditions of ill-health, produce suppurative inflammation without the association of microbia. If we base our treatment upon a mere supposition respecting the nature of a disease we must often be led into error.

But I am not sitting in judgment respecting methods of treatment; I am dealing with facts and elucidating them by comparisons, for upon these we must base both our faith and practice.

An illustration of the extreme chemico-vital treatment of disease as distinguished from the dynamic treatment advocated by Hahnemann is afforded by the administration of alkalies in order to increase the oxidation of fats in cases of obesity. Scurvy is believed to be induced by a deficiency of potash salts in the food, hence the remedy for scurvy would be to supply this deficiency; but it is found that these salts are not all that is required to re-establish the health. Rheumatism is believed to be prodnced by the formation of an excessive amount of lactic acid which is supposed to have an affinity for certain tissues and incites in them rheumatic inflammation and *alkalies* are given to neutralize this acid and protect the tissues. The dynamic treatment would restrict the excessive formation of lactic acid. It was formerly taught that when iron was given for "anemia," it was drawn by some occult process directly into the blood current to make up the deficiency existing there, but it is now known that iron salts cannot be absorbed and taken into the blood. Dr. Simon Baruch says (*Medical Record*, June 3d, 1893, p. 695): "The idea that mineral agents can be directly supplied to the blood is exploded. The iron contained in the human system only amouuts to from fifteen to forty-eight grains, and in the worst cases of anemia the amount of iron lost is only from three to four grains. This quantity can be furnished by a single pound of good beef," hence it is not the mere presence of iron in the blood that is needed but its dynamic influence.

Hahnemann reached the same conclusions nearly a century ago.

It is like fighting the wind to be aiming at pathological conditions in disease. We must select an agent which will influence the vital principle, so that more healthy action may be attained when we treat the sick.

From the internal administration of materials called drugs we are able to produce a *series* of influences upon the organism, influences dependent upon :

*First*—The physical properties of the drug.

*Second*—Physiological influences, and

*Third*—Dynamic or curative effects.

For instance, if we should administer a mass of silver nitrate, we get its mechanical effects, if it is large, hard, and irregular in outline; its chemical effects resulting in the superficial destruction of tissue; its physiological effects upon the kidneys, liver, and heart to produce fatty degeneration; its curative, dynamic effect, when we apply it to ulcerative surfaces as an excitant of the tissues to a healthier action, or when we give it in small doses to check the pain and vomiting of chronic inflammation or chronic ulceration of the stomach as recommended by Ringer, and to relieve flatulent dyspepsia, purulent ophthalmia, etc., as is done by the homœopathists.

In order to obtain the curative aid it is capable of affording, it must not be used in large masses but dilute so as to avoid the manifestation of its physical, chemical, and purely physiological effects.

The same is true of all drug action.

By something more than a coincidence during Hahnemann's time or toward the close of the last century the human mind reached the great principle of the indestructibility of matter. Prof. E. L. Youmans, in an article entitled, "A Terse Statement of the Doctrine of Forces," says: "What the intellectual activity of ages failed to establish by all the resources of reasoning and philosophy was accomplished by the invention of a mechanical implement, the balance of Lavoiser. When nature was tested in the chemist's scale it was first found that never an atom is created or destroyed; that though matter changes form with protean facility, traversing a thousand cycles of change, vanishing, and reappearing incessantly, yet it never wears out or lapses into nothing. This age will be memorable in the history of science for having demonstrated that the same great principle applies also to *forces*, and for the establishment of a new philosophy concerning their nature and relations. Heat, light, electricity, and magnetism are now no longer regarded as substantive and independent existences, subtile fluids with peculiar properties, but simply as modes of motion in ordinary matter, forms of energy, which are capable of mutual conversion, imponderable, invisible, and indestructible. Heat is a mode of energy, manifested by certain effects. It may be transformed into electricity, which is another form of force producing different effects." Magnetism resulting from an induced current of this electricity passing near a piece of soft iron is another form of energy, all subtile, invisible, and imponderable, governed by a law characterized by Faraday as "the highest in physical science which our faculties permit us to perceive, the most far-reaching principles that adventuring *reason* has discovered in the universe," yet one which the physician is dealing with each day he lives, to prescribe for human ills.

"Its stupendous reach spans all orders of existence. Not only does it govern the movements of the heavenly bodies, but it presides over the creation of the constellations; not only does it control those radiant floods of power which fill the eternal spaces, bathing, warming, illumining, and vivifying our planet, but it rules the actions and relations of men, and regulates the march of earthly affairs. Nor is its dominion limited to physical phenomena; it prevails equally in the world of mind, controlling all the faculties and processes of thought and feeling, originating all physiological phenomena by which our organisms are kept alive. The star suns of the remoter galaxies dart their radiations across the universe; and, although the distances are so profound that hundreds of centuries may have been required to traverse them, the impulses of force enter the eye, and, impressing an atomic change upon the nerve, give origin to the sense of sight. Star and nerve-tissue are parts of the same system. Stellar and nerve forces are correlated—nay, nerve sensation awakens thought and kindles emotion, so that this wondrous dynamic chain binds into living unity the realms of matter and mind through measureless amplitude of space and time."

Each drug plant or chemical product we use has stored within it its particular dynamic force. If we liberate it, it is not wasted, but under favorable conditions it is correlated in the human organism. To make use of this force we must control it so that it can be received into the organism in the most suitable manner. Thus we extract the active principle from the medicinal plant or finely divide the chemical product. The gross formation contains it, but this is only the storehouse; and to those of us who need that which is within, it is but a hindrance and obstacle which we have no need of, except to preserve the rich treasure until such time as we may call it forth for use. The division of matter does not destroy the material nor the force that dwells within. If you break the magnetic needle in twain, each part is a magnet still; and if you sever it into a thousand parts, each part will arrange itself under favorable conditions so that it points unerringly north and south. What a lesson we can learn from these things! We are as dumb animals if we see nothing beyond the gross materialistic form of objects; and yet all these higher realities are but faint glimpses which science has obtained in the dim dawn of discovery of the glories of a future existence. They are but pebbles gathered from the shores of the great ocean of truth compared with the mysteries still hidden in the bosom of the mighty unexplored.

I shall not enter into a discussion of the homœopathic law of cure; I want to allude only to the homœopathic method of using medicines which is closely allied to the law. I want to elucidate the idea that the curative properties of medicines may be developed by the process of trituration, succussion, and division. Within certain limits this division of matter makes them more potent as curative agents, empowering them to act in conjunction with the dynamic, invisible, imponderable, vital force within the human organism without hindrance. We aim to eliminate from drug action the physical, chemical, and physiological effects by division of matter. Drugs are then said to be dynamized. The extent to which the division of matter is to be carried in order to eliminate these effects and develop the dynamic power is a point which must be left for experience to determine. As matter is never lost nor force annihilated in matter, it makes but little difference, perhaps, if only the process is carried to the extent of the elimination of all the effects the *material* of the drug is capable of producing except the *curative dynamic influence.* The system is least burdened by the smallest curative dose, and overdosing by the too frequent repetition of the medicine is to be guarded against as much as overdosing by the administration of too large a quantity of the drug.

